# Development an Inflammation-Related Factor-Based Model for Predicting Organ Failure in Acute Pancreatitis: A Retrospective Cohort Study

**DOI:** 10.1155/2021/4906768

**Published:** 2021-09-10

**Authors:** Yunpeng Peng, Xiaole Zhu, Chaoqun Hou, Chenyuan Shi, Dongya Huang, Zipeng Lu, Yi Miao, Qiang Li

**Affiliations:** ^1^Pancreas Center, First Affiliated Hospital of Nanjing Medical University, 300 Guangzhou Road, Nanjing, 210029 Jiangsu Province, China; ^2^Pancreas Institute, Nanjing Medical University, Nanjing, 210029 Jiangsu Province, China

## Abstract

Several inflammation-related factors (IRFs) have been reported to predict organ failure of acute pancreatitis (AP) in previous clinical studies. However, there are a few shortcomings in these models. The aim of this study was to develop a new prediction model based on IRFs that could accurately identify the risk for organ failure in AP. *Methods*. 100 patients with their clinical information and IRF data (levels of 10 cytokines, percentages of different immune cells, and data obtained from white blood cell count) were retrospectively enrolled in this study, and 94 patients were finally selected for further analysis. Univariate and multivariate analysis were applied to evaluate the potential risk factors for the organ failure of AP. The area under the ROC curve (AUCs), sensitivity, and specificity of the relevant model were assessed to evaluate the prediction ability of IRFs. A new scoring system to predict the organ failure of AP was created based on the regression coefficient of a multivariate logistic regression model. *Results*. The incidence of OF in AP patients was nearly 16% (15/94) in our derivation cohort. Univariate analytic data revealed that IL6, IL8, IL10, MCP1, CD3+ CD4+ T lymphocytes, CD19+ B lymphocytes, PCT, APACHE II score, and RANSON score were potential predictors for AP organ failure, and IL6 (*P* = 0.038), IL8 (*P* = 0.043), and CD19+B lymphocytes (*P* = 0.045) were independent predictors according to further multivariate analysis. In addition, a preoperative scoring system (0-11 points) was constructed to predict the organ failure of AP using these three factors. The AUC of the new score system was 0.86. The optimal cut-off value of the new scoring system was 6 points. *Conclusions*. Our prediction model (based on IL6, IL8, and CD19+ B Lymphocyte) has satisfactory working efficiency to identify AP patients with high risk of organ failure.

## 1. Introduction

Generally, acute pancreatitis (AP) commonly presents as a mild and self-limiting course with slighter clinical symptoms. However, nearly 20% AP patients present with severe disease, which is correlated with higher rates of organ failure and mortality [1, 2]. According to previous studies, organ failure typically not only develops early in the course of acute pancreatitis but also may develop later due to infected pancreatic necrosis-induced sepsis. Organ failure is the most important determinant of outcome in acute pancreatitis [3]. Therefore, early identification of potential risk factors for OF occurrence in AP patients is helpful for physicians to select accurate and effective treatment interventions.

In previous studies, many factors have been reported as independent risk factors for the development of OF in AP, such as lipase, albumin, BUN, and pleural effusion. Moreover, the role of inflammation-related factors (IRFs) in secondary OF is getting more and more attention [4]. The severity of AP is significantly associated with the occurrence and development of systemic inflammatory response syndrome (SIRS). In the early stage of acute pancreatitis (AP), abundant cytokines induced by local pancreatic inflammation enter the bloodstream and further cause SIRS by “trigger effect,” which eventually leads to organ failure [5]. Different immune cells and cytokines are the key members, and abnormal alteration of some members has been confirmed as potential indicators for the progression of AP. For example, lower level of CD3+ CD4+ T lymphocytes and higher level of CD19+ B lymphocytes could predict organ failure in the early phase of AP [6], and escalating levels of cytokines like IL6, IL8, MCP-1, and TNF-a were frequently observed in AP patients with organ failure [7]. Furthermore, the most commonly used inflammation biomarkers in clinical practice, C-reactive protein (CRP) and PCT, play similar roles in the early prediction of AP-related OF [8, 9]. It has been reported that patients with early elevated levels of CRP and PCT tended to suffer with organ failure in several studies [10].

In general, various types of IRFs were potential predictive indicators of OF in patients with AP. However, the forecast performance of each indicator was insufficient in clinical practice. Therefore, the purpose of this work was to develop and validate a simple and effective risk model based on IRFs for the early recognition of OF.

## 2. Patients and Methods

Consecutive patients with AP treated in the First Affiliated Hospital of Nanjing Medical University (Nanjing, Jiangsu province, China) from 2017.06 to 2018.06 were retrospectively enrolled in our study, and patients referred from other hospitals were excluded in our study. Three criteria were applied to diagnose AP, including persistent abdominal pain, serum amylase or lipase elevation of more than 3 times upper limit of normal, and characteristic ultrasound and/or CT findings. Patients were diagnosed as AP when two or more signs mentioned above were observed. Furthermore, patients with recent surgery (less than 1 week), chronic, traumatic, endoscopic, or recurrent acute pancreatitis, immunosuppression, or immune deficiency were excluded. The principles of the Helsinki Declaration were applied in the performance of our study, and this study was approved by Ethics Committee of the First Affiliated Hospital of Nanjing Medical University.

According to the modified Marshall scoring system, serum creatinine was applied to define renal failure (more than 1.9 mg/dL), systolic blood pressure was used to define cardiovascular failure (less than 90 mmHg), and ratio of PaO2/FiO2 was used to confirm respiratory failure (less than 300 mmHg). The duration was used to distinguish transient OF (≤48 h) from persistent OF (>48 h) [11]. Patients who have persistent OF (>48 h) was finally enrolled into this study.

### 2.1. Data Collection

The peripheral blood samples of all patients were obtained within 24 h after admission to hospital. Peripheral blood lymphocyte subset assay was applied to detect the percentage of CD3+T lymphocytes, CD3+ CD4+T lymphocytes, CD3+ CD8+ cytotoxic T lymphocytes, CD16+ CD56+ natural killer cells, and CD19+ B lymphocytes. Blood routine examination was used to obtain white blood cell count and percentage. Blood samples were routinely collected on AP patients admitted to our hospital and then stored and subsequently analyzed for the concentration of 10 cytokines (IL1a, IL1b, IL4, IL6, IL8, IL10, IL13, MCP-1, IFNg, and TNFa) by using the Human Inflammation Array Q1 (QAH-INF-1-1) (RayBiotech, Norcross, America). Moreover, other IRF (CRP and PCT) data, several biochemical parameters, and basic clinical information were also collected.

### 2.2. Statistical Analysis

All statistical analyses were conducted by Stata/SE version 10.0 for Windows. Descriptive data were presented as mean ± standard deviation for quantitative variables or absolute and relative frequencies for qualitative variables. Student's *t*-test or nonparametric tests (Chi-square and Mann–Whitney) were used to compare OF and NOF group. Firstly, the analysis of univariate regression was conducted to evaluate the association between the predictive factors and OF. Second, factors with a *P* < 0.1 in the univariate regression analysis were included in multivariate logistic regression analysis. A *P* value of 0.05 were deemed to be statistically significant. The logistic regression coefficients as well as the allocation of scoring points for each predictive factor, based on the regression coefficients, are given in Table 1. Our calculation of IRF model is based on the disease risk prediction model of the Framingham Heart Study [12]: (1) estimation of the regression coefficients of the multiple logistic regression; (2) organize the risk factors into meaningful categories and determine a reference value (midpoint) for each category, continuous variables were divided into groups, and the group median value was used as the reference value. Then, we need to determine the basic risk reference value of all risk factors. The basic risk reference value refers to that if a certain risk factor of the patient takes this value, the risk score is 0. The higher the value is, the higher the score is, and the higher the risk is. (3) Calculate the distance (*D*) between the classification of each risk factor and the basic risk. (4) We set the unit distance (*B*) for 1 point. (5) Each risk factor was scored, point = *D*/*B*.

## 3. Results

### 3.1. Characteristics of Enrolled Patients

A total of 100 patients were enrolled in this study, 94 patients were finally enrolled in the statistical analysis, and 6 patients were excluded due to the lack of IRF data (3 patients for PCT, 1 patient for CRP, 2 patients for PCT and CRP). Related information of clinical parameters was shown in Table 2, including age, gender, etiology, number of OF, length of hospital stay, hospital cost, APACHE II score, RANSON score, and IRFs levels. As described in Table 2, biliary pancreatitis was the most common type of AP in this cohort (54/94); the incidence of persistent OF was nearly 16% (15/94). Another 5 patients had transient organ failure and were placed in the control group.

### 3.2. Univariate and Multivariate Analysis Based on Clinical Information and IRFs

All patients enrolled in final analysis were divided into two groups according to the occurrence of OF (NOF vs. OF). Univariate analysis was selected to evaluate the association between OF occurrence and IRF levels (Table 3). The analytic data demonstrated that the levels of cytokines IL6, IL8, IL10, and MCP1 were abnormally increased in OF group, and the variation tendency of CD19+ B lymphocytes, PCT, APACHE II score, and RANSON score were similar to these cytokines. However, the percentages of CD3+ CD4+ T lymphocytes were inversely decreased in OF group than that in NOF group. The other IRF levels were not markedly correlated with the presence of OF. These results indicated that IL6 (OR 1.01, 95% CI 1.004-1.024, *P* = 0.003), IL8 (OR 1.20, 95% CI 1.08-1.33, *P* = 0.001), IL10 (OR 1.26, 95% CI 1.08-1.47, *P* = 0.003), MCP1 (OR 1.01, 95% CI 1.003-1.026, *P* = 0.004), CD3+ CD4+ T lymphocytes (OR 0.92, 95% CI 0.87-0.98, *P* = 0.01), CD19+ B lymphocytes (OR 1.11, 95% CI 1.04-1.18, *P* = 0.001), PCT (OR 1.14, 95% CI 1.04-1.26, *P* = 0.006), APACHE II score (OR 1.28, 95% CI 1.05-1.55, *P* = 0.015), and RANSON score (OR 3.57, 95% CI 1.86-6.85, *P* < 0.001) were potential predictors for OF in AP.

To assess whether the potential risk factors (*P* < 0.01) were independently associated with the progression of OF, multivariate logistic regression analysis was subsequently conducted. The related results suggested that IL6 (OR 1.10, 95% CI 1.001-1.03, *P* = 0.038), IL8 (OR 1.28, 95% CI 1.01-1.63, *P* = 0.043), and CD19+ B lymphocytes (OR 1.13, 95% CI 1.003-1.275, *P* = 0.045) were independent predictors for OF development, as well as RANSON scores (OR 9.38, 95% CI 1.90-46.39, *P* = 0.006) (Table 4).

**Figure 1:**
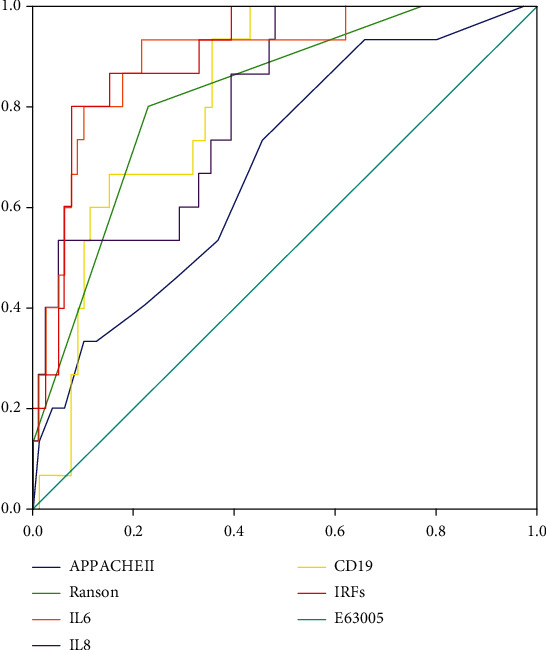
ROC curve analysis of the IRFs and relative clinical factors in prediction of OF.

ROC curve analysis were applied to evaluate the predictive value of IRF model for OF, as well as APACHE II score and RANSON score. As shown in Table 5 and Figure 1, the AUC of IRFs model was 0.905 while the AUCs of APACHE II, RANSON, IL6, IL8, and CD19+ B lymphocytes, respectively, were 0.684, 0.821, 0.895, 0.808, and 0.821. In total, the AUC of IRFs model was higher compared to APACHE II and RANSON score, as well as individual factor enrolled in IRF model.

Therefore, these three inflammation-related variables (IL6, IL8, and CD19+ B lymphocytes) were finally selected to develop a prediction model (IRFs model). All details of IRF model are shown in Table 1, including classification method, regression coefficient, and assigned score for each predictive factor.

### 3.3. Point Allocation for Predictors of Organ Failure Based on Regression Coefficients

**Figure 2:**
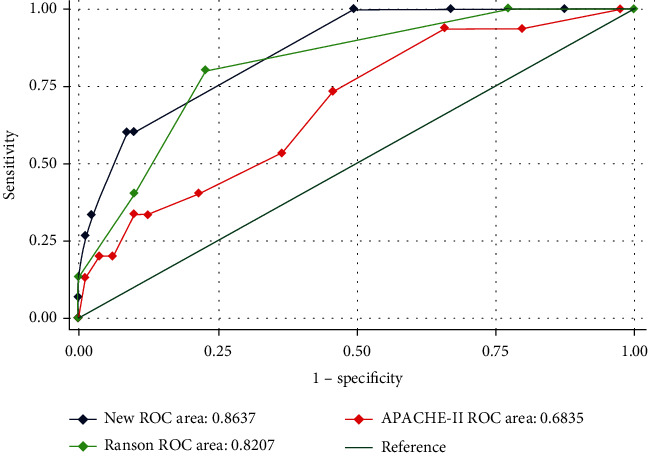
Comparisons of the AUCs between scoring systems in prediction of OF.

Results of multivariate logistic regression analysis suggested that IL6, IL8, and CD19+ B lymphocytes are independent predictors for organ failure. Therefore, these three factors were selected into the final model for the construction of the prediction score (IRF score). The prediction scores based on this model range from 1 to 11, and AP patients with higher score had increasing risk to suffer with OF. The risk of AP patients with OF progressively increased as the score increased. ROC curve analysis was applied to evaluate the predictive value of new score system for OF, as well as APACHE II score and RANSON score. The AUC 0.86 (95% CI: 0.78-0.94) of ROC analysis demonstrated a satisfactory discrimination of new score system and higher than other two criteria (APACHE II and RANSON score) (Figure 2). A score of 6 points was suggested to be the optimal cut-off value (Youden index = 0.511) to divide the risk strata.

**Figure 3:**
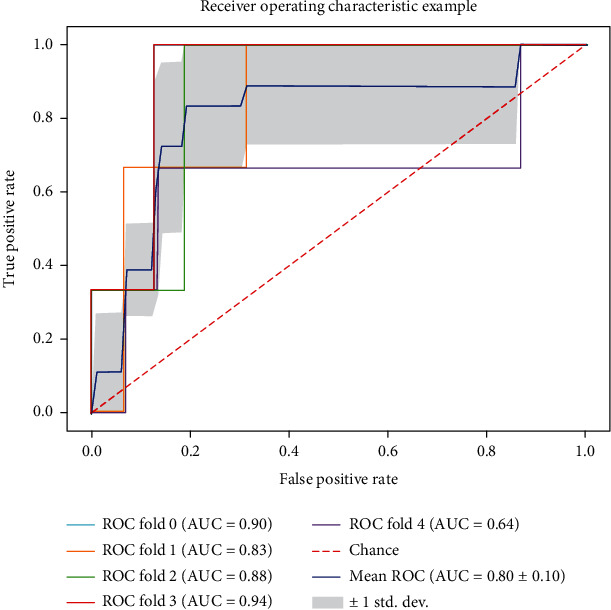
Mean receiver-operating characteristic (ROC) curve of the new prediction score for prediction of OF in tenfold crossvalidation.

The mean ROC curve of tenfold crossvalidation of the new prediction score (IRF score) is shown in Figure 3, which gave an AUC of 0.80 ± 0.10 (mean ± 1 std.dev.) indicating satisfactory separating capacity for our model.

## 4. Discussion

As reported in previous studies, organ failure was a common but dangerous clinical manifestations in AP, and organ failure was also regarded as the main reason for the death of AP patients. Therefore, the identification of individuals with organ failure in the early stage was the key step for the treatment of these patients. Different kinds of IRF-induced SIRS were reported to be responsible for the occurrence and development of organ failure in AP, and it has been confirmed that some of these IRFs could function as independent indicators for such pathogenetic process. Several researches suggested that abnormal alteration of some immune cells could play the predictive role in the organ failure process, such as CD4+ lymphocyte, CD19+ B lymphocyte, neutrophil-lymphocyte ratio, and CD14hiCD16-monocytes [6, 13, 14]. Some studies focused on cytokines demonstrated that various cytokine levels were significantly associated with the incidence of organ failure, including IL6, IL8, IL17, MCP-1, and TNF-a [15]. Moreover, the inflammatory factors PCT and CRP were also reported to work as predictors for the organ failure of AP [10, 16]. However, nearly all studies evaluated the predictive efficiency of potential factors, respectively, and the combined value of these factors for organ failure prediction was still to be investigated.

In our study, we firstly assess the predictive value of several IRFs for the development of organ failure in AP, including the percentages and/or cell counts of immune cells, the levels of 10 cytokines, and 2 common inflammation biomarkers. Similar to previous studies, the results based on this aspect suggested that the increased levels of IL6, IL8, and CD19+ B lymphocyte were, respectively, correlated with the increased incidence rate of organ failure [6]. IL6 and IL8 were frequently used cytokines which could closely participate in the differentiation of certain immune cells. For example, IL6 was the key accelerator in the final differentiation of B-cells into Ig-secreting cells, and IL8 had the function on neutrophil activation [17]. IL6 and IL8 could take part in a wide variety of inflammation-associated disease by inducing the inflammatory response, such as rheumatoid arthritis [18], ulcerative colitis [19], and acute pancreatitis [20]. In acute pancreatitis, the levels of these cytokines in peripheral blood were abnormally increased and significantly associated with the disease development. And some basic studies focused on AP pathogenesis also found that IL6 and IL8 could function as the important medium for different incentives induced and aggravated inflammatory process [21]. In addition, activated CD19+ B lymphocyte could also influence the process of various inflammatory diseases by promoting antigen-mediated humoral immunity [22]. By comprehensively considering the important roles of these three factors in inflammatory process and our analytic data, we selected them to further conduct prediction model.

In clinical practice, two AP scoring systems, APACHE II and RANSON, were widely used to evaluate to the severity of such disease. In fact, these two models were relatively cumbersome due to the excessive parameters enrolled. Therefore, researchers specialized in AP have been trying to build simple and feasible models to replace them. In recent years, several simple models have been developed to evaluate the severity of acute pancreatitis in previous investigations. For example, Hong et al. published a prediction model for severe acute pancreatitis by using SIRS, albumin, BUN, and pleural effusion [23]; Qiu et al. conducted a model for the prediction of multiple organ failure in moderately severe and severe acute pancreatitis by using HCT, K-time, IL-6, and creatinine [24]; intra-abdominal pressure and body mass index were applied to establish prediction model to evaluate the AP severity [25]. However, there was no similar model based on IRFs (promote the inflammatory process in AP) ever published. In our study, we initiatively conducted an effective model for the prediction of AP-related organ failure by using three IRFs, including IL6, IL8, and CD19+ B lymphocyte. This model was more simple and convenient than the widely accepted AP points-scoring systems (APACHE II and RANSON), and the AUCs, sensitivity, and specificity of this model were similar to others, sometimes even better. This shows that the prediction efficiency of our model was partially better than existing points-scoring model. On the other hand, we can use this simple model to identify AP patients with high risk of organ failure rapidly and effectively by only detecting IL6, IL8, and CD19+ B lymphocyte.

However, our study also has some limitations. Firstly, there were data missing in this study, especially the information of some other IRFs related to AP which were not enrolled, such as IL17, IL12, and M2 monocyte, which might be responsible for the selection bias. Secondly, all data applied in our study were collected retrospectively, which might reduce the reliability of results based on these data. Lastly, because of the funding problem, the sample size of our study was insufficient, not only the total number of all AP patients but also the number of AP patients with organ failure, and the validation cohort for IRF model was also absent. In the future research, we will expand the sample size and set up a validation cohort to further confirm the working efficiency of our prediction model, as well as enroll more potential IRFs.

## 5. Conclusions

In conclusions, IL6, IL8, and CD19+ B lymphocyte were reliable predictors for the organ failure of AP, and the prediction model has satisfactory working efficiency to identify AP patients with high risk of organ failure. This study may improve clinical prevention and treatment strategies of AP progression.

## Figures and Tables

**Table 1:** Predictive scoring system for OF.

Preoperative factor		*β* coefficient	Points contributed
IL 6 (pg/mL)	<7	0	0
	7-150	0.015257141	1
	150-250		3
	>250		4
IL 8 (pg/mL)	<8.1	0	0
	8.1-21.3	0.248266704	2
	>21.3		4
CD19+ B lymphocyte s(%)	<9	0	0
	9-14	0.122927762	1
	>14		3

**Table 2:** Characteristics of enrolled patients.

Variable	NOF (*n* = 79)	OF (*n* = 15)	*Z*	*P*
Age (years) (mean ± SD)	50.7 ± 16.3	47.1 ± 18.2		0.774
Sex, male/female	33/46	7/8		0.821
Time of onset (hours)				0.705
0-24 h	28	7		
24-48 h	27	4		
48-72 h	24	4		
Etiology				0.417
Biliary, *N* (%)	43	11		
Alcohol, *N* (%)	8	2		
Hypertriglyceridemia, *N* (%)	13	1		
Other, *N* (%)	15	1		
APPACHE II score	3.8 ± 2.5	5.7 ± 3.2		0.011
RANSON score	1.1 ± 0.9	2.3 ± 9.8		*<0.001*
Length of hospital stay (days)	9 (7, 12)	16 (15, 36)	-4.06	*<0.001*
Hospital cost (CNY∗)	32435 (22203, 41689)	75075 (62198, 174771)		*<0.001*
Inflammatory markers (pg/mL)	Median (P25, P75)	Median (P25, P75)		
IL1a	2.35 (1.55, 4.25)	1.55 (1.28, 2.56)	-1.76	0.474
IL1b	3.32 (1.73, 8.18)	3.16 (1.69, 11.38)	-0.03	0.895
IL4	2.33 (1.42, 4.75)	2.08 (1.57, 3.67)	-0.49	0.431
IL6	24.43 (10.18, 42.3)	106.9 (78.97, 188.13)	-4.82	*<0.001*
IL8	4.52 (2.99, 7.86)	14.14 (5.68, 18.03)	-3.76	0.002
IL10	1.51 (0.83, 2.62)	8.25 (4.33, 14.98)	-4.67	0.003
IL13	0.58 (0.32, 0.76)	0.31 (0.2, 0.65)	-1.36	0.348
MCP1	152.64 (125.18, 192.46)	234.11 (165.02, 280.94)	-1.01	0.001
IFNg	1.56 (0.93, 2.49)	1.21 (0.78, 2.24)	-2.71	0.505
TNFa	5.78 (2.34, 11.16)	4.4 (2.2, 11.55)	-0.45	0.624
PCT (ng/mL)	0.42 (0.12, 1.18)	2.07 (0.33, 23.38)	-2.65	*<0.001*
CRP (mg/mL)	90 (40.1, 90)	90 (84.9, 90)	-1.02	0.84
Routine blood test				
WBC, ×109/L (mean ± SD)	10.7 ± 4.1	13.3 ± 4.9		0.244
LY, ×109/L median (P25, P75)	1.13 (0.85, 1.57)	0.99 (0.85, 1.25)	-1.172	0.241
MO, ×109/L median (P25, P75)	0.53 (0.44, 0.78)	0.45 (0.34, 0.95)	-0.749	0.454
NE, ×109/L (mean ± SD)	8.7 ± 3.9	11.4 ± 4.4		0.161
Immunity markers (%)				
CD3 T lymphocytes	67.8 ± 9.5	58.6 ± 14.1		0.002
CD3CD4 T lymphocytes	39.8 ± 10.0	31.3 ± 8.3		0.007
CD3CD8 cytotoxic T lymphocytes	23.06 (17.56, 28.53)	21.56 (13.21, 26.84)	-1.1	0.272
CD16+ CD56+ natural killer cells	10.9 (6.7, 18.39)	14.67 (7.63, 17.39)	-0.49	0.624
CD19+ B lymphocytes	14.5 (10.2, 19.8)	25.57 (17.9, 29.97)	-3.929	*<0.001*
CD4CD8 cytotoxic T lymphocytes	1.76 (1.29, 2.24)	1.55 (1.09, 2.87)	-0.083	0.934

∗CNY (Chinese Yuan), white blood cell counts (WBC), lymphocyte (LY), neutrophils (NE), and monocyte (MO).

**Table 3:** Univariate analyses of factors predicting OF.

	Odds ratio	Std. err.	*z*	*P* > ∣*z*∣	95% conf. interval
Age	0.9868881	0.0171669	-0.76	0.448	0.9538086, 1.021115
Gender	0.8198758	0.4637188	-0.35	0.725	0.2705899, 2.484189
APACHE II score	1.275259	0.1280648	2.42	0.015	1.047415, 1.552667
RANSON score	3.570297	1.186867	3.83	<0.001	1.860978, 6.849637
IL1a	0.9204542	0.1064778	-0.72	0.474	0.7337286, 1.154699
IL1b	0.9975549	0.0183145	-0.13	0.894	0.9622973, 1.034104
IL4	0.9486596	0.0635995	-0.79	0.432	0.8318493, 1.081873
IL6	1.014311	0.0049215	2.93	0.003	1.004711, 1.024003
IL8	1.200382	0.0638983	3.43	0.001	1.081455, 1.332386
IL10	1.263606	0.0983619	3.01	0.003	1.084806, 1.471875
IL13	0.5039705	0.3617765	-0.95	0.34	0.123415, 2.057986
MCP1	1.011875	0.0041537	2.88	0.004	1.003767, 1.020049
IFNg	0.8752206	0.1774049	-0.66	0.511	0.5882761, 1.302129
TNFa	0.9844869	0.0314253	-0.49	0.624	0.9247815, 1.048047
PCT	1.144502	0.0563309	2.74	0.006	1.039253, 1.260409
CRP	1.01183	0.0111544	1.07	0.286	0.990202, 1.03393
Cause	0.6276013	0.1894851	-1.54	0.123	0.3472878, 1.13417
CD3 T lymphocytes	0.9298539	0.0238692	-2.83	0.005	0.8842285, 0.9778336
CD3CD4 T lymphocytes	0.9241535	0.0283805	-2.57	0.01	0.8701696, 0.9814864
CD3CD8 cytotoxic T lymphocytes	0.9575243	0.0332156	-1.25	0.211	0.8945867, 1.02489
CD16+ CD56+ natural killer cells	1.021315	0.032097	0.67	0.502	0.9603039, 1.086201
CD19+ B lymphocytes	1.110688	0.0364459	3.2	0.001	1.041504, 1.184468
CD4CD8 cytotoxic T lymphocytes	1.050004	0.2806481	0.18	0.855	0.6218407, 1.772975
WBC, ×109/L	1.078487	0.0699162	1.17	0.244	0.9498018, 1.224606
LY, ×109/L	1.516559	0.9935976	0.64	0.525	0.4199339, 5.476934
MO, ×109/L	0.3913318	0.253425	-1.45	0.147	0.1099791, 1.392451
NE, ×109/L	1.101204	0.0761925	1.39	0.164	0.961552, 1.261138
Ca	0.0473802	0.0504324	-2.86	0.004	0.0058825, 0.3816217

**Table 4:** Multivariate analyses of factors predicting OF.

	Odds ratio	Std. err.	*z*	*P* > ∣*z*∣	95% conf. interval
RANSON score	9.380673	7.650668	2.74	0.006	1.896762, 46.39328
PCT	1.099312	0.0612663	1.7	0.089	0.9855586, 1.226196
IL6	1.015374	0.0074628	2.08	0.038	1.000852, 1.030107
IL8	1.281802	0.1574817	2.02	0.043	1.007494, 1.630794
IL10	1.056482	0.1449462	0.4	0.689	0.8073833, 1.382434
MCP1	0.982185	0.0102192	-1.73	0.084	0.9623586, 1.00242
CD19+ B lymphocytes	1.130803	0.069377	2	0.045	1.002684, 1.275292

**Table 5:** predictive value of IRFs model and other clinical factors for OF.

Preoperative factor	AUC	Std. err.	95% conf. interval	
			Lower limit	Upper limit
APACHEII	0.684	0.072	0.541	0.826
RANSON	0.821	0.055	0.712	0.929
IL6	0.895	0.044	0.808	0.981
IL8	0.808	0.056	0.699	0.916
CD19+ B lymphocytes	0.821	0.047	0.729	0.913
IFRs	0.905	0.036	0.835	0.976

## Data Availability

All data in our study are available from the corresponding authors upon reasonable request.
